# Green clues: unveiling the role of bryophytes in forensic science

**DOI:** 10.1093/fsr/owaf026

**Published:** 2025-11-10

**Authors:** Jenna Merkel, Matt von Konrat, Lloyd R Stark, Andrew Laurence, Laura Briscoe, Becky Collings, Peter Carrington, Danny Kreider, Juan Larraín, Alan Lichamer, Gary Merrill, Anton Reznicek, R Jan Stevenson, Frank W Telewski, J B Wells

**Affiliations:** Department of Forensic Sciences, George Washington University, Washington, DC; Botanical Collections, The Field Museum, Chicago, IL; School of Life Sciences, University of Nevada, Las Vegas, NV; U.S. Customs and Border Protection, Department of Homeland Security, Chicago, IL; Botanical Collections, The Field Museum, Chicago, IL; Cryptogamic Herbarium, New York Botanical Garden, Bronx, NY; Botanical Collections, The Field Museum, Chicago, IL; Forest Preserves of Cook County, River Forest, IL; W. J. Beal Botanical Garden, Michigan State University, East Lansing, MI; Botanical Collections, The Field Museum, Chicago, IL; Botanical Collections, The Field Museum, Chicago, IL; Centro de Investigación en Recursos Naturales y Sustentabilidad (CIRENYS), Universidad Bernardo O’Higgins, Santiago, Chile; Botanical Collections, The Field Museum, Chicago, IL; Botanical Collections, The Field Museum, Chicago, IL; Centro de Investigación en Recursos Naturales y Sustentabilidad (CIRENYS), Universidad Bernardo O’Higgins, Santiago, Chile; Department of Integrative Biology, Michigan State University, East Lansing, MI; Department of Plant Biology, Michigan State University, East Lansing, MI; Ludington Police Department, Ludington, MI

**Keywords:** forensic sciences, bryophytes, forensic botany, forensic bryology, mosses

## Abstract

Forensic botany is the use of plant material as evidence to aid in forensic investigations. Bryology is the study of bryophytes, which consist of mosses, liverworts, and hornworts. Botanical evidence as a whole, and more specifically potential bryophyte evidence, is an underused potential tool that can provide crucial information in criminal investigations. This paper (i) outlines a review of all bryophyte applications to forensic science, to the best of our knowledge, (ii) demonstrates the potential of using this type of evidence, (iii) presents details into each case, and (iv) highlights the various uses of bryophytes as forensic evidence. From our review, all cases have seemingly been limited to mosses. The overarching goal is for this review to be a resource that encourages law enforcement to search for plant fragments and microscopic bryophytes.

**Key Points**
- Bryophytes are an underutilized potential tool within forensic science.
- Bryophytes are ubiquitous as well as have a number of unique features and can be found in almost every environment around the world.
- Many reported and published case reports exemplify the numerous applications for bryophytes and other botanical evidence within forensic science.

## Introduction

Forensic botany, the use of plant evidence in criminal investigations, is an underutilized yet valuable tool in forensic science. Plant material can provide critical links between a victim, suspect, and crime scene, but its potential has been largely overlooked, with few professionals trained to properly identify and collect botanical samples [1]. This gap in knowledge and application represents a significant opportunity for enhancing forensic investigations, particularly with the expanded use of non-flowering plants such as bryophytes. This paper briefly opens with the historical development of forensic botany, its current applications, and the potential of bryophytes as a powerful resource in forensic science. This review will focus on mosses and their underexplored potential in forensic science. Mosses, together with liverworts and hornworts, are commonly referred to as bryophytes, all of which share similar characteristics. Although much forensic botanical work has centered on flowering plants and algae such as diatoms, bryophytes have several characteristics that make them particularly suited for forensic investigation, including their ubiquity, resilience, and ability to persist in various environments. We will summarize the known applications of bryophytes in forensic cases and explore their potential for wider use in law enforcement investigations. All cases that we have found are limited to mosses only.

## Forensic botany

The use of plants in forensic investigations is an underused tool that has proved effective in establishing links and associations, as previously stated, and also in providing other crucial information that has potentially been overlooked. There has been a very limited number of cases reported using botanical evidence and even fewer law enforcement personnel that are trained to identify and properly collect these types of samples [2]. One of the first accounts of forensic botany being used was in 1932 in the Lindbergh kidnapping case when wood evidence was presented and accepted in the case hearing [3]. This case is said to be the birth of forensic botany and highlighted the importance and usage of different aspects of plant specimens as evidence in a criminal case and represented one of the first major uses of forensic wood analysis in a US criminal trial [4]. Testimony helped establish the credibility and value of trace and material evidence, particularly wood, in criminal forensics. While this was the first highly publicized case that utilized botanical evidence, the use of plants in criminal cases dates back to the early 1900s as outlined by Hans Gross in his pioneering textbook on criminal investigations [5]. The recognition of botanical evidence is the first step, followed by the proper documentation, collection, and most importantly preservation of samples in order to remain viable for downstream laboratory analysis. There are many aspects of plant samples that can be useful in providing information to a case including plant structure, the anatomy of a plant, and the environments which they inhabit [6]. There have been several significant publications documenting and focusing on botany in forensic science including Heather Coyle’s *Forensic Botany: Principles and Applications to Criminal Casework*, providing comprehensive reviews and applications to criminal casework [1]. Since its emergence, many different disciplines of forensic botany have been formed and researched in order to establish an application within forensics. Forensic botany has included many disciplines, including ecology, limnology, palynology, molecular biology, anatomy, and morphology [4, 7–10], highlighting the need for greater awareness and training within law enforcement. Forensic botany has been popularized by the BBC television series, *Silent Witness*, focusing on forensic pathologists, with an episode that even included the moss *Weissia rostellata* (Brid.) Lindb. Forensic scientists in the episode used the presence of the moss to strengthen circumstantial evidence by showing that the suspect had been in a particular location.

The most commonly used and validated discipline is forensic palynology, the use of microscopic pollen grains and spores within forensic applications. Palynology was first used in forensics in the 1950s and proved valuable due to the persistence of pollen on various items including but not limited to clothing, shoes, and hair [11]. Pollen is resilient to various environmental conditions due to the structure and composition of its outer layer, making pollen applicable for forensic purposes since there is a high possibility of being intact when they are recovered [12]. Pollen grains can be identified by their different shapes, sizes, and orientations and are unique to each plant species. In forensics, the successful identification of pollen recovered can reveal information on a specific location or environment from which the sample originated, or reconstruct its travel history [11]. U.S. Customs and Border Protection was the first US agency to develop a forensic palynology team that utilizes pollen to track illegal drugs, provide investigative leads for unidentified children, and help with issues of national security by performing geolocation [13]. The Bella Bond case is a great example of palynology being applied to forensic investigations. This case, outlined by Laurence and Bryant, occurred in 2015, where the remains of a baby girl were found in a black trash bag on the shore of the Boston Harbor [14]. They reported extracting pollen grains from the clothing and hair of the deceased child, sorting and counting the grains, identifying the pollen present, and then formulating the most likely area she came from. The pollen analysis provided crucial information that led to the identification of the victim and the arrest of suspected individuals, proving its relevance and highlighting the importance of its use in criminal investigations. While there are many other disciplines and subdisciplines that may be further explored, the main focus of this review is the potential application of bryophytes to forensic science.

### Bryology

Bryology is the study of bryophytes which are small, non-flowering, spore-producing green land plants that lack a lignified vascular system [15]. For many years, bryophytes were classified as three independent plant divisions including Bryophyta (mosses), Marchantiophyta (liverworts), and Anthocerophyta (hornworts) [16]. However, recent studies indicate support for de-ranking the hornworts, liverworts, and mosses, classifying the bryophytes as a whole group under the division Bryophyta, and recognizing the three classes Anthocerotopsida, Marchantiopsida, and Bryopsida [17]. Together, bryophytes are the second largest group of land plants after flowering plants, with an estimated 18 000 to 20 000 species [18], and are pivotal in our understanding of early land plant evolution [19, 20]. To our knowledge, all forensic application of bryophytes has been limited to mosses. While bryophytes share a number of features such as lacking lignified vascular tissue, each of the three groups have their own unique characteristics and properties that separate them [21]. Bryophytes are an important component of diversity in many ecosystems worldwide, particularly in forests, mountains, wetlands, and tundras [15, 22, 23]. They are particularly common in wet environments due to a number of their physiological (e.g., relying on surface absorption for water) and morphological properties (e.g., their simple form, with root-like structures called rhizoids for anchorage as well as simple leaf-like structures). Yet, bryophytes also exhibit a high desiccation tolerance and have the ability to survive harsh environmental conditions [24], e.g., the desert moss *Syntrichia caninervis* Mitt. has been reported to survive a thermal tolerance of >120 °C, representing a record for all adult eukaryotes [25, 26]. Mosses have even been recorded to survive through *in situ* cryptobiosis after six centuries of glacier burial [27].

Bryophytes are of great ecological and environmental significance, playing an important role as possible indicators of climate change [28, 29], in nutrient cycling [30], and through their water retention—reducing soil nutrient loss and flooding risk [31]. Bryophytes also form minute “forests” and provide the matrix that structures communities inhabited by microscopic life forms such as tardigrades, mites, rotifers, micro-mollusks, microalgae, microfungi, cyanobacteria, diatoms, single-celled eukaryotes, and numerous groups of invertebrates [32, 33]. Bryophytes, and mosses in particular, have been shown to be effective low-cost biological monitors of metal air pollution because they readily accumulate pollutants over time, potentially reflecting long-term pollution levels [34, 35].

Traditional and ethnic uses of bryophytes that have been used for many different applications throughout human history have been extensively reviewed [36–38]. For instance, the Native American tribes were known to use various moss species to treat burns, bruises, and other injuries by grinding them into a paste-like consistency [39]. Bryophytes have been known to exhibit antibiotic and antiviral properties which have proved useful within medical practices and research [40]. *Sphagnum* moss has been observed to display both of these properties and has been effective against microorganisms for antibiotic abilities as well as antiviral effects through the production of humic acid [40]. Bryophytes have also been used in many household items such as stuffing and filling for pillows, bedding, upholstery, and dolls as well as decorations [41]. Interestingly, bryophytes are well known for containing chemicals with interesting biological properties such as antibacterial, antifungal, cytotoxic, insect anti-feedant, and muscle-relaxing activity [42, 43].

### Potential attributes of bryophytes for forensic application

Bryophytes offer an expanded botanical resource for forensic botany, which has traditionally focused on flowering plants. Their potential attributes make bryophytes particularly valuable in forensic investigations for numerous reasons. Bryophytes are ubiquitous, i.e., they are commonly found in many habitats, including urban areas, which makes them essential pieces of a larger puzzle when conducting a forensic investigation, yet also allow for the collection of reference samples [44]. Furthermore, bryophyte fragments and shoots are easily detached or broken off and can become attached to items such as shoes, clothing, or vehicles and can exist in samples of soil, dirt, or other debris [44]. These fragments or shoots could be helpful in establishing associations between an individual and a crime scene [2]. Bryophytes are extremely resilient and are able to survive and thrive in adverse and various environmental conditions, which adds to their potential forensic application. Bryophyte fragments are easily preserved if collected and packaged properly, allowing for future analyses to be performed. All of these characteristics are beneficial for bryophytes to be used to supplement forensic investigations and provide crucial information about events that may have occurred and to establish links between potential suspects, victims, and the crime scene. Medina [45] summarized the unexpected uses of mosses in forensics, emphasizing some features that make them suitable for investigative applications.

**Figure 1:**
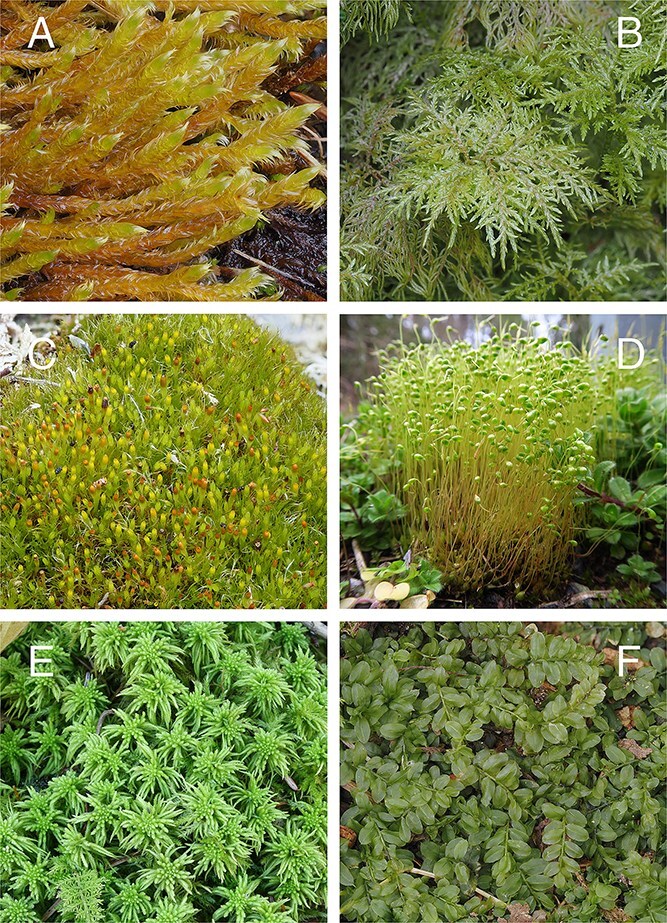
Plant growth habits of mosses encountered in forensic contexts outlined in this review illustrating the variation in growth patterns and the presence of sporophytes (reproductive features that include a seta (or stalk) and a capsule that produces spores). (A) *Calliergonella lindbergii* (Mitt.) Hedenäs, creeping habit, sparsely branched; (B) *Hylocomium splendens*, regularly, profusely branched, with feather-like branches in appearance; (C) *Tetraplodon angustatus* with sporophytes, cushion-forming habit with densely packed, erect stems and numerous red-brown capsules on slender setae; (D) *Funaria hygrometrica* with sporophytes, forming loose tufts with erect, simple stems and curved capsules on elongated setae; (E) *Sphagnum girgensohnii*, forming dense cushions with clustered branch systems and distinctive star-like branch arrangements; (F) *Plagiomnium ciliare,* with broad, flattened leaves and creeping to semi-erect growth habit*.* Photo credits: Blanka Aguero.

**Figure 2:**
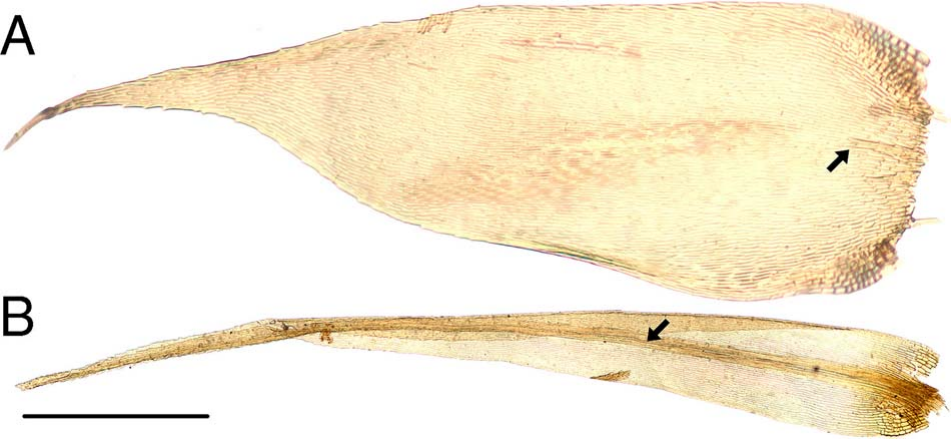
Whole leaves illustrate variation in leaf shape, and the presence or absence of a distinctive midrib; useful features in moss identification. (A) *Hypnum cupressiforme* showing a broad, acuminate leaf (leaf tip tapering gradually to a sharp point) with a very short, double nerve (arrow) [C1048566F], scale bar = 300 μm; (B) *Dicranum scoparium* showing a narrowly triangular leaf, with a very well-developed nerve (arrow) [C1055519F], scale bar = 300 μm.

**Figure 3:**
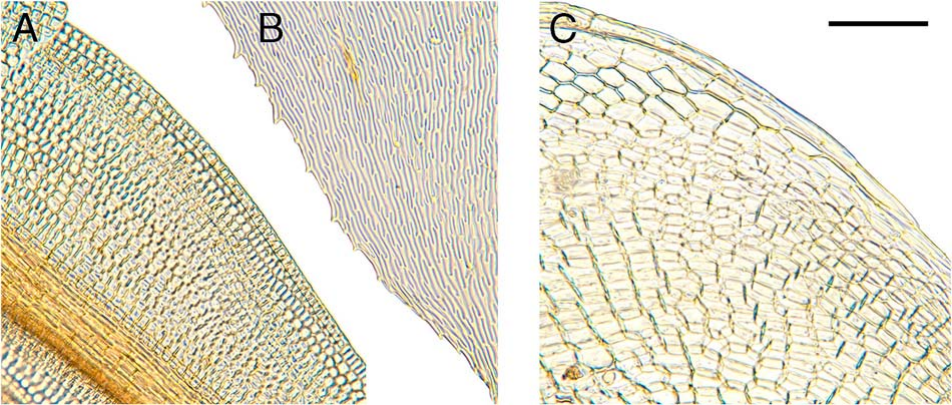
Diversity of leaf margins at high magnification, showing some useful morphological features. (A) *Ceratodon purpureus*, entire and recurved margin with cells somewhat different from the rest of the leaf blade [C1044845F]; (B) *Hylocomium splendens*, with an irregularly toothed margin; (C) *Ptychostomum capillare* (Hedw.) Holyoak & N. Pedersen., entire margin, with a distinctive row of differentiated, long cells [C0074109F], scale bar = 100 μm.

**Figure 4:**
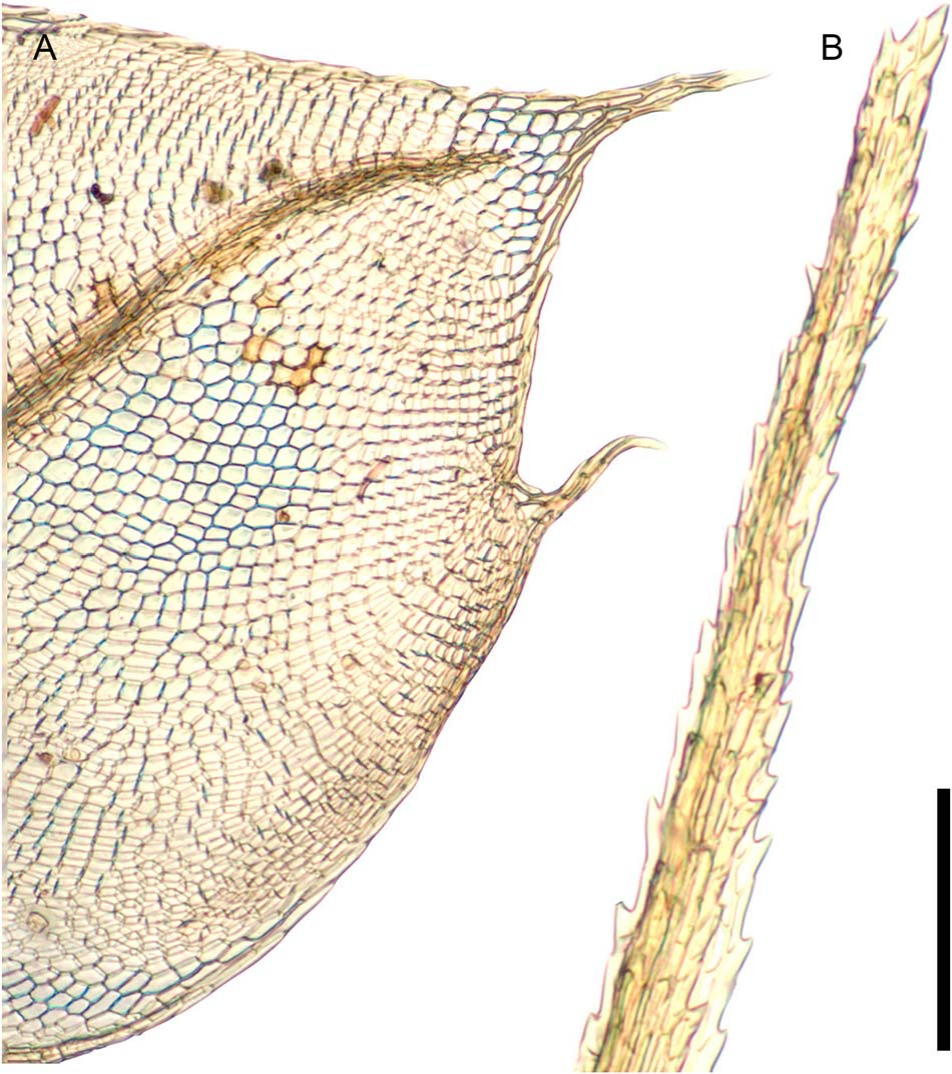
Leaf apices, key to distinguishing moss taxa in fragmentary samples. (A) *Ptychostomum capillare*, an acuminate apex, with the nerve ending just below it [C0074109F]; (B) *Dicranum scoparium*, leaf tip, long, strongly serrated (or toothed) apex [C1055519F], scale bar = 200 μm.

**Figure 5:**
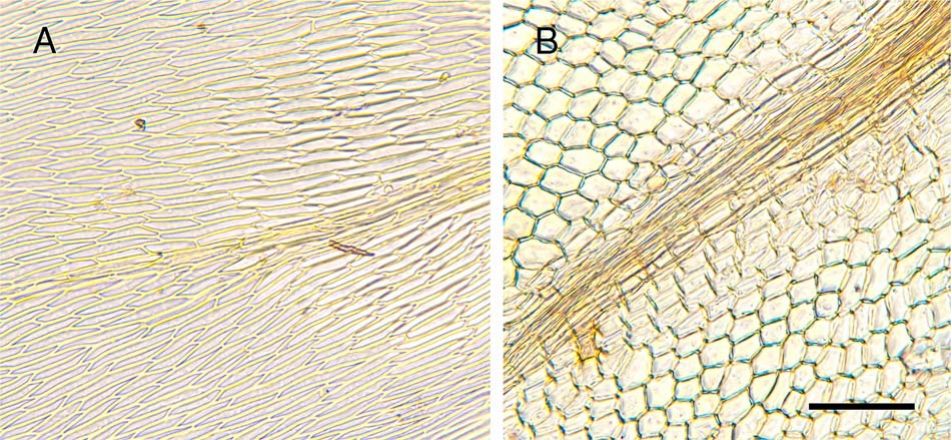
Leaf cell structure, highlighting the unique areolation patterns useful in microscopic identification. (A) *Brachythecium albicans* with long narrow cells [C2025573F]; (B) *Ptychostomum capillare* with subquadrate to hexagonal-shaped cells [C0074109F], scale bar = 100 μm.

**Figure 6:**
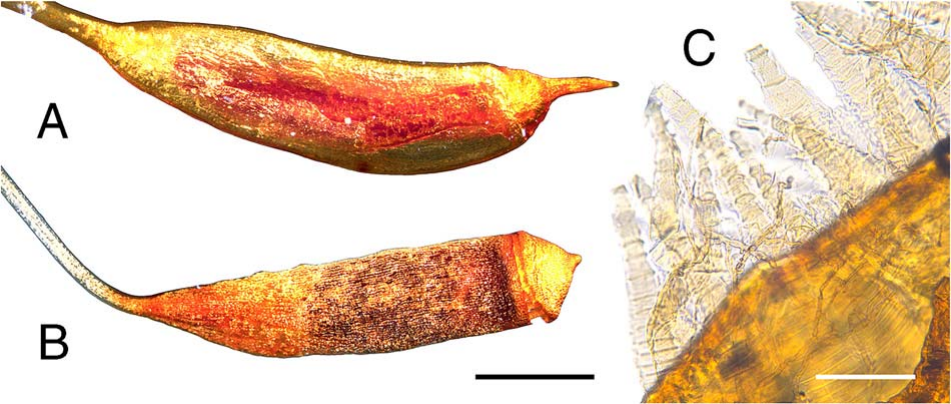
Sporophyte traits, including capsule shape and peristome teeth, which are taxonomically informative and can persist as forensic evidence. (A, B) Portion of seta and capsule with an operculum (lid). (A) *Hypnum cupressiforme*, capsule with a rostrate lid (beaked) [C1048566F], scale bar = 1 mm; (B) *Ptychostomum capillare*, capsule with a nipple-like lid [C0074109F], scale bar = 1 mm; (C) peristome teeth, *H. cupressiforme* [C1048566F], scale bar = 100 μm.

In addition, identification often only requires a small portion of the sample to provide enough information to accurately identify the main group in which a specimen belongs [38]. There are a number of morphological features exhibited by mosses that taxonomists utilize to aid identification (Figures 1–6). These include plant habit (Figure 1), leaf shape (Figure 2), leaf margin and apices (Figures 3 and 4), leaf anatomy and cell size and shape (Figure 5), and features associated with the sporophyte—the capsule phase of a bryophyte that produces spores (Figure 6). Despite the small size of individual stem mosses, they can form relatively conspicuous mats and in some environments form a dominant part of the vegetation. Furthermore, even with only a tiny fragment of a leaf, one can often surmise the type of habitat. Figure 1 also showcases a diverse range of moss genera that have been used in forensic cases, illustrating variations in morphology from the delicate, feathery fronds of *Hylocomium splendens* (Hedw.) Schimp. (Figure 1B) to the robust, densely packed clusters of *Sphagnum girgensohnii* Russow (Figure 1E).

The use of molecular tools has also revolutionized our understanding of species concepts. This is exemplified by a study that designed species-specific microsatellite markers to establish a link between the bryophyte evidence recovered from an investigation to reference samples collected from the crime scene [46]. In that study, researchers evaluated the effect to which bryophytes can be used for forensics. In order to do this, they designed an experiment to determine how well plant fragments attach to shoes after being exposed to a forested environment. Twenty hours were spent hiking through a forest and the final 4 h were spent hiking on gravel or paved roads. They found that bryophyte material was found on 28% of shoes, recovering 22 shoots from five different species. This supports the idea that bryophytes are easily attached to items, specifically shoes, and can remain after hours of continuous walking and movement. They also explored if bryophyte DNA remains viable when exposed to the elements for an extended period of time. Fresh samples were stored in paper bags and left in a shed in southern Finland for 18 months, exposed to temperatures ranging from −28 °C to 25 °C. They were successfully able to obtain genotyping results from these samples which confirms the reliability of DNA sequencing for recovered bryophyte fragments [46].

The persistence of fragmentary material is an important feature of mosses that might be useful for forensic science. For example, a study described the observation of 200-year-old shoots of the epiphytic moss *Brotherella henonii* (Duby) M. Fleischer discovered in a bark pocket where shoots of the moss were gradually enclosed in the tree trunk together with the bark during subsequent growth [47]. Some moss species, e.g., *H. splendens*, exhibit distinct annual growth increments that appear as clearly demarcated tiers or segments along their main stems [48, 56]. This has been very clearly described, discussed, and illustrated [48]. Each tier represents 1 year’s growth, formed as the moss produces new shoots seasonally while the previous year’s growth becomes dormant, creating visible boundaries between growth periods. This tiered architecture functions similarly to tree rings, providing a chronological record that can be counted backwards from the growing tip to determine the age of each segment. In forensic contexts, this growth pattern enables investigators to establish potential timelines by examining moss growing on or around evidence [56]. The applications of moss growth-rate analysis in forensic timeline reconstruction are detailed below.

## Reported applications of bryophytes to forensic science

As far as we know, there has been no single source summarizing the application of bryophytes in forensics. Table 1 reflects all known forensic cases involving bryophyte material, to the best of our knowledge, after a review of the literature, the World Wide Web, and consultation with the bryological community. It is apparent that there are a seemingly limited number of media news reports, popular articles, community newsletters, and peer-reviewed publications highlighting bryophytes as a potential resource as an aid for criminal investigations. Yet, Table 1 indicates that bryophytes have been broadly used within forensic investigations. Bryophytes have proved useful for homicides, missing persons, postmortem interval (PMI) calculations, suicides, and desecration cases (Table 1). These are outlined in detail below. However, this may not be the full extent of the use of bryophytes when applied to criminal investigations. In addition to Table 1, there are some other anecdotal examples from the bryological community where moss was used within investigations but lacked substantial evidence to be used for conviction—demonstrating the potential of using botanical evidence to enhance investigations.

**Table 1:** Reported forensic cases involving bryophytes (mosses).

**Year**	**Location**	**Type of case**	**Species utilized**	**Description/application**	**Source**
1929	Tyrol, Austria	PMI	*Funaria hygrometrica* Hedw. *Ceratodon purpureus* (Hedw.) Brid. *Tetraplodon angustatus* (Hedw.) Bruch & Schimp.	Used growth rate of mosses growing on a decomposing human skeleton to determine the PMI	[53]
2001	Finland	Homicide	*Brachythecium albicans* (Hedw.) Schimp. *Calliergonella lindbergii* (Mitt.) Hedenäs *Ceratodon purpureus* (Hedw.) Brid.	Used recovered bryophyte fragments to link suspects to the scene of the crime where human remains were discovered	[58]
2006	Stockholm, Sweden	PMI	*Hylocomium splendens* (Hedw.) Schimp. *Dicranum scoparium* Hedw. *Brachythecium* Schimp.	The shoot structure of bryophyte fragments was used to calculate the PMI of recovered remains	Personal comm. with Lars Hedenäs
2008	Portugal	Missing person	*Ptychostomum capillare* *Hypnum cupressiforme* Hedw. *Bryum* sp. *Campylopus flexuosus* (Hedw.) Brid. (or related species) *Campylopus introflexus* (Hedw.) Brid.	Used bryophytes to calculate PMI to determine if remains could belong to a missing individual who disappeared 6 years prior	[57]
2009	Alsip, IL, USA	Desecration—removal of buried remains	*Fissidens taxifolius* Hedw.	Bryophyte fragments that were buried with discarded remains that had been dug up and removed from grave sites were used to determine how long the remains had been buried to establish a timeline for investigators	See Case Report in this issue
2010	Perugia, Italy	PMI	*Leptodictyum riparium* (Hedw.) Warnst.	Used the growth rate of bryophytes covering remains to calculate the PMI and determine the age of the remains	[55]
2011	Ludington, MI, USA	Abduction and homicide	*Sphagnum affine* Renauld & Cardot*/Sphagnum palustre* L. *Sphagnum girgensohnii* Russow*/Sphagnum fimbriatum* Wilson *Plagiomnium ciliare* (Müll. Hal.) T.J. Top. *Dicranum flagellare* Hedw. *Hypnum* sp. Brachytheciaceae	Recovered bryophyte fragments from the suspect were used to try to locate an area where all species may grow together in order to locate the body of a 4-month-old baby after her disappearance	[59]
2011	Not reported	PMI	*Brachythecium rutabulum* (Hedw.) Schimp.	Growth rates of similar bryophyte species were used to calculate the PMI	[36]
2013	Italy	PMI	*Hygrohypnum luridum* (Hedw.) Jenn.	Used bryophytes growing on skull and clothes to calculate PMI and date the remains	[55]
2015	Siena, Tuscany, Italy	Suicide	*Tortula muralis* Hedw. *Ptychostomum capillare*	Used bryophytes to reconstruct the crime scene and determine sequence of events	[2]
2005	Taipei, China	Suicide	Not identified	Used bryophytes to determine most likely scenario to differentiate between suicide and homicide	[64]

PMI: postmortem interval.

The following cases are organized primarily by case type (homicide, suicide, etc.), though it should be noted that PMI estimation is a cross-cutting technique that appears in multiple case categories. Where PMI estimation is the primary application, this is noted in the case descriptions.

### PMI estimation

Bryophytes have proved useful in establishing a PMI for recovered human remains and has been the most common forensic application of bryophytes. The PMI refers to the amount of time since the death of an individual and provides crucial data used in the medicolegal death investigation and can be used to establish links within a case [49]. The task of estimating the PMI has proven to be quite challenging and slightly unreliable due to the numerous factors that affect the rate of decomposition of a body [49]. Once an individual dies and the body functions stop working, the body undergoes changes known as algor, rigor, and livor mortis. Algor mortis refers to the change in the body’s temperature due to the lack of heating and cooling mechanisms [50], rigor mortis refers to the stiffening of the body due to a lack of ATP, a unit of energy, within the body’s cells [51], and livor mortis refers to the discoloration of the skin due to the pooling of blood within the body [51]. These are all physical changes that can be observed within the first 72 h of death and occur at different rates depending on the environmental temperature, conditions, and other outside factors acting on the corpse [49]. Forensic entomology can also be useful in estimating the PMI by observing the types of insect species that begin to colonize the body, the rate of which they appear, their activity, and their stage of development [52]. Bryophytes can be used to reinforce the weight behind the PMI estimation by calculating their growth rate as described above.

While not a forensic case, the bog bodies that have been discovered throughout Europe are a great indicator of how bryophytes can slow the decomposition of a body. The Tollund Man, one of the most well-preserved bog bodies discovered, was found in 1950 in the Bjældskovdal bog in Denmark [53]. Bog bodies are human remains that have been naturally mummified as a result of the conditions of the bog. Due to the lack of drainage in the bog and the layers of *Sphagnum* moss that grow, the growth of bacteria and accumulation of other organic material that usually results in proper breakdown and decomposition of a body is greatly decreased [54]. This shows the importance of moss accumulation on human remains and how it may impact the state of skeletonization that can therefore influence a PMI calculation and an investigation.

### PMI estimation using moss colonization time

#### Pioneering case: moss on human remains, Austria (1929)

*Case location and details*—An early case reported in a German book by Kiebacher et al. describes heavily decomposed human remains covered in moss, recovered in Tyrol, Austria, near Winkleralm in 1929 [53]. The remains were discovered during an investigation and presented an opportunity to apply bryological methods to forensic science.

*Species involved*—Helmut Gams at the University of Innsbruck identified small amounts of Funaria hygrometrica Hedw. (Figure 1D) and *Ceratodon purpureus* (Hedw.) Brid. (Figure 3A), as well as a large colony initially thought to be *Tayloria rudolphiana* (Garov.) Bruch & Schimp. After further analysis, the sample was identified as *Tetraplodon angustatus* (Hedw.) Bruch & Schimp. (Figure 1C) [53].

*Forensic significance*—Using what they described as developmental time, i.e., the period required for the mosses to colonize and grow to the observed extent, Gams estimated the time since the body was deposited. Kiebacher reported that *T. angustatus* (Hedw.) Bruch & Schimp. is likely to grow on decomposing bones [53], supporting its potential forensic value in future postmortem investigations.

#### Moss growth on a boot and PMI, Westchester, New York, USA (1980s)

A personal communication from Dr. William R. Buck and Dr. Spencer J. Turkel informed the authors of the current paper about a case involving the Metropolitan Forensic Anthropology Team and the Westchester Medical Examiner, in the 1980s. A moss was found growing on a boot, found nearby, of a man’s body in a wetland of Westchester, New York. Dr. William R. Buck was shown the boot with a substantial amount of moss growing on it, including with sporophytes, and estimated the moss had been growing for at least 2–3 years. This aided law enforcement who wanted to know how long the body might have been there, e.g., months or years *versus* decades, in order to determine identification. The account was published in [65, 66], as an abstract, and confirmed by the first author; however, we were unable to see this publication firsthand.

#### Moss evidence in a cemetery investigation, Chicago, Illinois, USA (2009)

In 2009, employees of the Burr Oaks Cemetery in Chicago, Illinois, were accused of digging up the remains of individuals buried at the cemetery and reselling the burial sites [67]. Samples of mosses were found buried with recovered discarded human remains and a team at the Field Museum, Chicago, USA, identified the moss as *Fissidens taxifolius* Hedw. They were also able to analyze various aspects of the moss fragments and determined that they had been buried no longer than 24 months, which contradicted the suspect’s timeline and placed the accused at the scene of the crime. While not a homicide or missing-persons case, the moss evidence provided crucial information that led to the successful conviction of guilty individuals that may not have been as efficient without it. This case is described in further detail as a case report in a later issue of this journal [67].

### PMI estimation using moss growth rate

#### A case from Stockholm, Sweden (2006)

*Case location and details*—Another case involving the PMI was reported by Lars Hedenäs (personal communication) and took place in Stockholm, Sweden, in 2006. Human remains were discovered in the city, partially covered by a tarp. The police had no leads on the identity of the individual, and estimating the length of time the body had been at the location was considered essential for narrowing down a potential time frame and the search radius for identifying the deceased.

*Species involved*—To assist with this, mosses growing on the body and the tarp were collected and analyzed. The mosses were identified as *H. splendens* (Hedw.) Schimp. (Figures 1B, 3B), *Dicranum scoparium* Hedw. (Figures 2B, 4B), and a species of *Brachythecium* Schimp. Lars Hedenäs used the shoot structure and anatomical characteristics of the reproductive organs of each moss to assess their age individually. Based on this analysis, *H. splendens* (Figure 1B) was determined to be ~8–9 years old, *D. scoparium* was estimated at 5–6 years, and the *Brachythecium* species was ~2 years old.

*Forensic significance*—From these moss age estimates, the police concluded that the remains likely belonged to a man who had been missing for at least 10 years. This case provided compelling support for the forensic application of bryology, demonstrating how moss development can offer a reliable means of estimating the PMI when other evidence is lacking.

#### A forensic case study from Italy (2010)

*Case location and details*—Reported by Lancia et al. [55], human remains were discovered in a wooded area near the town of Perugia, Italy, in 2010. Analysis of the remains indicated that the deceased was an elderly Caucasian female, ~5′2″ (1.5748 m) in height. In order to aid identification, it became crucial to estimate the time of death and how long the body had remained at the site since the individual had passed away.

*Species involved*—Bryophytes were found colonizing the skull and were identified as *Leptodictyum riparium* (Hedw.) Warnst. Lancia et al. [55] aimed to use the growth data of this moss to calculate the PMI. However, no established growth-rate data for *L. riparium* were available at the time. Instead, the team relied on comparative data from *Hypnum cupressiforme* Hedw. (Figures 2A, 6A, 6C), a morphologically similar and well-documented species. By counting the number of segments along the moss stems, they estimated the age of the mosses to be between 20 and 30 months.

*Forensic significance*—This moss age estimate enabled investigators to narrow the search to individuals who had gone missing at least 2.5 years prior [5]. They were able to identify an 80-year-old woman who had disappeared in June 2007, which was further confirmed through radiological examinations of the skeletal remains with those performed when she was still alive. The case highlights how bryophyte growth patterns, even when direct species-specific data are lacking, can still be applied through comparative analysis to provide a reasonable estimate of the PMI.

#### A forensic case report by Ann Mills (2011)

*Case location and details*—In 2011, Ann Mills reported a forensic case to the bryophyte research community involving a human skeleton discovered at an outdoor site. The presence of moss growth prompted further investigation into the time since death [36].

*Species involved*—The moss was identified as *Brachythecium rutabulum* (Hedw.) Schimp. Responses from the bryological community provided useful information on its growth rate, ecological behavior, and suitability for estimating colonization age.

*Forensic significance*—As with similar cases described above, the primary goal was to use this information and analysis to estimate the age of colonization and thereby help determine how long ago the skeleton may have been discarded, providing a useful timeline for investigators [36].

#### Mosses that colonized a skull; reported by Caccianiga et al. (2021)

*Case location and details*—Caccianiga et al. [56] report on a 2013 case where a skull was discovered on a river bank in an Alpine valley in northern Italy. This was one of eight case reports involving botanical evidence used to enhance criminal investigations. The remains were analyzed alongside clothing found at the scene, providing important context for the forensic examination [56].

*Species involved*—The moss *Hygrohypnum luridum* (Hedw.) Jenn. was found colonizing both the skull and clothes. The recovered sample contained three fully intact branches as well as some decomposing branch material that provided crucial temporal information [56].

*Forensic significance—*Experts applied knowledge of this moss species’ growth rate to determine that each branch signified ~1 year, with the decomposing material indicating an additional year. This bryophyte-based estimate established a PMI of 4 years, which corroborated findings from dendrochronological analysis of shrub roots recovered from the same location [56].

#### Skeletonized remains in a Portuguese Forest; reported by Cardoso et al. (2010)

*Case location and details*—Cardoso et al. [57] describe a case from northern Portugal where skeletonized adult male remains were discovered in a wooded area. The remains exhibited significant plant colonization, suggesting extended environmental exposure prior to discovery. Investigators noted the presence of both moss species and shrub roots growing directly on the skeletal elements, prompting a detailed botanical analysis to establish a timeline [56].

*Species involved*—Five distinct moss species were identified growing on the skeletal remains: *Ptychostomum capillare* (Hedw.) Holyoak & N. Pedersen (as *Bryum capillare* Hedw.) (Figures 3C, 5B, 6B), *H. cupressiforme*, *Bryum* sp. Hedw., *Campylopus flexuosus* (Hedw.) Brid. (or related species), and *Campylopus introflexus* (Hedw.) Brid. Various shrub roots were also documented interacting with the remains, providing additional botanical evidence [57].

*Forensic significance*—Based on comprehensive analysis of the botanical evidence, experts determined a timeline of ~3 years for complete decomposition and skeletonization followed by an additional 3 years of plant growth. This 6-year PMI aligned with information about a 61-year-old man reported missing from the area during that timeframe. While considered circumstantial evidence for identification purposes, the bryophyte analysis provided investigators with crucial temporal context that supported other case findings [57].

### Homicide investigations

While PMI calculations are a useful application of these plant fragments to criminal investigations, bryophytes can be used for a much wider scope and can be incredibly helpful in establishing connections between unknown samples that are found and known reference samples that are collected at a specific location, the crime scene. This type of information can be crucial in homicide and suicide cases where it is unclear the manner of death and investigators must rely on evidence to tell a story.

#### DNA of moss samples links suspects to Finnish murder scene (2001)

*Case location and details*—Korpelainen and Virtanen [58] report the first documented use of bryophytes in criminal proceedings in 2001, >20 years ago [45]. The case involved a man whose body was discovered 5 km from a cafe where he was last seen meeting with three former criminal partners. These three individuals were subsequently arrested as suspects in connection with his death [58].

*Species involved*—Bryophyte fragments were recovered from the shoes and clothing of all three suspects, as well as from the vehicle they had driven. Three moss species were identified from these samples: *Brachythecium albicans* (Hedw.) Schimp. (Figure 5A), *Calliergonella lindbergii* (Mitt.) Hedenäs (Figure 1A), and *C. purpureus*. Reference samples of the same species were collected from the crime scene for comparative analysis [58].

*Forensic significance*—The recovered bryophyte fragments were compared to known samples from the crime scene to determine common origin and support investigative suspicions. DNA sequencing was performed on both known and questioned samples to confirm whether the genotypes were identical. This groundbreaking study pioneered the forensic application of bryophytes in murder investigations, demonstrating the evidential value of bryophytes and other clonal specimens to forensic science [58].

#### Katherine Phillips (Baby Kate); mosses, diatoms, and seed plants aid law enforcement in a murder investigation, Michigan, USA (2011)

*Case location and details*—A case that took place in 2011 known as the “Baby Kate” case used botanical evidence, including bryophytes to attempt to locate a body. This case has been widely reported in the popular media but never scientifically documented. Co-authors von Konrat, Briscoe, Larraín, Merrill, Reznicek, Stevenson, and Telewski were directly involved in the case led by co-author retired Detective J.B. Wells. Sean Phillips was accused of the murder of Katherine Phillips, a 4-month-old baby who became known as “Baby Kate”. One year after her disappearance, Phillips wrote in a letter that Katherine had died as a result of an accident and he put her to rest in a “peaceful place”, but investigators were unable to recover her body. However, they were able to identify and collect moss tissue that was stuck in dried mud to the bottom of Phillips’ shoe [59].

The Ludington Police Department and Mason County Sheriff’s Office called on the public and requested that any individuals with plant identification skills aid in searching for and locating the specimens that were recovered from the bottom of Phillips’ shoe [59]. In addition to the volunteer scientists, there were support personnel from the Mason County Sheriff’s Office, Ludington Police Department, Michigan State Police, and local fire departments. Assisting in all the logistical planning was the Emergency Manager for Mason County Liz Reimink. Each group was accompanied by one of the experts from the Field Museum, Ferris State University, University of Michigan, and Michigan State University.

*Species involved*—The bryophyte samples were sent to a team at the Field Museum where they were able to identify the six different species as *Sphagnum affine* Renauld & Cardot or *Sphagnum palustre* L., *S. girgensohnii* Russow (Figure 1E) or *Sphagnum fimbriatum* Wilson, *Plagiomnium ciliare* (Müll. Hal.) T.J. Kop. (Figure 1F), *Dicranum flagellare* Hedw., *Hypnum* sp. Hedw. (most likely *Calliergonella lindbergii* var. *americana* (Renauld & Cardot) J.J. Atwood & Brinda), and Brachytheciaceae (possibly *Brachythecium* sp. or *Eurhynchium* sp.). The Field Museum produced a plant identification guide for >80 volunteer scientists involved in an evidentiary field search to look for and collect reference samples that would be used for comparison.

Based on the evidence, the species found on the sole of the suspect’s shoes, the target area would have consisted of red pine (*Pinus resinosa* Sol. ex Aiton), white cedar (*Thuja occidentalis* L.), three specific sedges (*Carex atlantica* subsp. *capillacea* (L.H.Bailey) Reznicek, *Carex leptalea* Wahlenb., and *Carex interior* L.H.Bailey), as well as *Sphagnum* moss—all growing within a close proximity, which is not a common occurrence according to Dr. Frank W. Telewski, a botanist from Michigan State University, and also co-author of this paper [60].

*Forensic significance*—The goal of using these bryophyte evidence samples, along with other plant specimens that were recovered, was to pinpoint an area where these species exist together which may have provided a potential location of Katherine’s body. Following a 2-day search, only two grids were found to support all the specimens in the same area: an area labeled “Green Road” and an area labeled “Lemke Drain”.

Telewski’s conclusions were supported by analyses of diatoms found on the sole of the suspect’s shoes. Existing data for diatoms in regional habitats were used to calculate indicators of environmental conditions by Dr Stevenson at Michigan State University, a co-author on this review, as is commonly done in water quality assessments [61–63]. These indicators showed the sediments came from a wetland habitat with relatively low pH and high nutrient concentrations. A wetland with low pH and high nutrients was rare in regional databases and pointed to Lemke Drain as a likely site for further investigation because the stream-side wetlands at Lemke Drain are located near a large agricultural field that could be a source of nutrients. Follow-up collections of sediments from Lemke Drain showed a higher similarity to diatoms on the suspect’s shoes than any other site in the regional database, indicating that Lemke Drain was a likely source of sediments on the suspect’s shoes.

Ludington Police Chief Mark Barnett stated that “The location that would support all these elements is so rare and specific to the surrounding area that it is only a matter of time before the exact location is found”. Barnett added “locations showing a high probability of containing all of these elements have been established and need to be searched to either confirm or eliminate whether Sean was there” [60]. This quote directly explains how this evidence was being used by the county police to try to find Katherine’s remains. While Katherine’s body was not recovered, the analysis and identification of the botanical samples recovered from Phillips’ shoes greatly narrowed down investigators’ search radius. A subsequent conversation between Detective J.B. Wells (co-author on this paper) and Sean Phillips (who was found guilty of second-degree murder) led to Sean Phillips pointing to an area on a map centered around Lemke Drain. This was an area that combined the same composition of diatoms, mosses, sedges, and trees that in all likelihood narrowed down the final resting place of Katherine Phillips from seven Counties to 50 square feet (4.65 m^2^).

### Suicide investigations

In terms of using bryophyte evidence in suicide cases, the known reports described them as pieces of a complicated puzzle that, when put together, helped investigators to determine what occurred and what could have been the most likely manner of death. In the following two cases, the moss found on the victim and in the surrounding crime scene provided information to reconstruct what investigators believed to have been the timeline of events leading to the victims’ deaths that involved no camera footage and no eye witnesses.

#### Moss reveals a suicide in Taipei, China (2005)

*Case location and details*—The case occurred in Taipei, China, in 2005 where an adult male was found hanging from a tree [64]. The initial investigation needed to determine if the death was a suicide or homicide.

*Species involved*—The specific moss species was not identified in the case report. The moss was found as smears on the inside of the victim’s wrists and was also growing on the tree from which the man was hanging [64].

*Forensic significance*—The moss evidence provided crucial information to determine the manner of death. Investigators found that the moss on the victim’s wrists matched the moss growing on the tree branch. This indicated that the victim acquired the moss smears while tying the rope around the branch himself, supporting the conclusion of suicide rather than homicide. This evidence eliminated the need for further criminal investigation [64].

#### Moss evidence reconstructs fatal fall sequence at an Italian mall

*Case location and details*—The case took place in Italy and involved a young woman who died after falling from a mall terrace [2]. Investigators were tasked with determining whether she jumped voluntarily or was pushed to her death. The body was discovered at the base of the building, and forensic teams conducted a thorough examination of both the victim and the entire path she would have taken to reach the terrace, including stairwells and walkways.

*Species involved*—Two moss species were identified from moss fragments: *Tortula muralis* and *Ptychostomum capillare*. These moss fragments were recovered from the bottom of the victim’s shoes and the masonry parapet of the terrace. The same species were found growing along the pathways leading to the fatal fall location. No date was provided for when this took place.

*Forensic significance*—After analyzing the crime scene, investigators found moss growing along the pathways leading to the terrace that appeared to have been walked on. The same moss species were found on both the victim’s shoes and the terrace parapet. This evidence suggested that she collected moss on her shoes while walking the stairwell and walkways, then climbed onto the parapet, depositing moss traces before jumping [2]. This moss transfer pattern strongly supported the theory of suicide over homicide, as it demonstrated that she voluntarily climbed onto the parapet rather than being pushed or thrown [2]. Without comparing these moss samples to establish the connection between locations, investigators would not have been able to confidently determine the manner of death. The case highlights how bryophyte fragments can attach to individuals and serve as microscopic evidence in reconstructing event sequences, providing critical forensic information that might otherwise be unavailable.

## Limitations and challenges

Despite their forensic potential, bryophytes have practical limitations. Identifying tiny fragments is often difficult and requires specialized expertise not necessarily available during investigations. Many investigators may not recognize bryophyte material as significant evidence or recognize a bryophyte fragment as being from a bryophyte. While moss growth rates can assist with PMI estimates, they vary greatly with temperature, moisture, and season [57]. Moreover, reliable growth data exist for only a few species and not all bryophytes have growth markers. The scarcity of trained bryologists also limits timely forensic consultation and practical application.

### Other biotic and abiotic microscopic evidence to consider

Trace evidence refers to microscopic evidence that is used within criminal investigations [68]. These elements require careful identification, collection, analysis, and comparison to provide information that is useful to solve [68]. Trace evidence is extremely beneficial in investigations because while individuals may believe that no evidence has been left behind, they may not realize the microscopic particles that they are leaving behind that will aid investigators. Common microscopic evidence that is used includes but is not limited to explosive and gunshot residue, tool marks, fibers, glass fragments, and hair. Other evidence such as plant material as highlighted in this paper along with seeds, insects, animal products, soil, sand, minerals, plastics, adhesives, lubricants, and cosmetics can also provide helpful insights [68].

Another group of organisms, lichens, although phylogenetically unrelated to bryophytes, representing two major kingdoms of life, often share similar habitats and are functionally related as the main hosts for cryptobiotic communities. Similar to mosses, they are incredibly resilient and are able to survive an array of conditions which contributes to their ability to accumulate on many different types of substrate, including human bone [69]. García et al. [69] report a case where multiple species of lichen were found growing on human remains that were recovered from the Deseado Massif in the Santa Cruz Province, Argentina. Seven species from 63 samples were identified, with *Caloplaca* s.l. and *Lecanora* s.l. being common on bones and potentially applicable for forensic investigations through growth-rate analysis to determine PMIs [70]. For wider application, more studies on lichen growth rates are needed, though their frequent occurrence on bones shows promise for enhancing forensic work. The discovery of both moss and lichen growing on whale vertebrae in Antarctica demonstrates how combining these organisms could lead to the possibility of their utility in forensic investigations involving bones.

Bryophytes create microhabitats supporting diverse microbiota including diatoms—unicellular, photosynthetic eukaryotes with indestructible siliceous cell walls that persist after death [68]. With ~15 000 established species found in nearly all aquatic environments, diatoms have forensic applications, particularly in aiding the diagnosis of drowning deaths, as they do not naturally occur in the human body but indicate water involvement [69]. Their forensic utility has been extensively documented [71–80, 82]. Moss-dwelling diatom communities vary based on environmental factors like nutrient concentrations, pH, salinity, water flow velocity, and temperature, enabling identification of specific habitats and potentially specific sites.

For example, one study with 101 moss samples found 191 distinct diatom taxa in the collections [81]. Prof. Stevenson, a co-author on this paper, also used diatoms as evidence as part of the Katherine Phillips case briefly outlined above. Given the diverse habitats of diatoms and their persistence in various environments, their applications in forensic science could potentially extend beyond drowning cases.

### Key findings, strategic recommendations, actionable insights, and future perspectives for enhanced forensic applications

Bryophytes (mosses, liverworts, and hornworts) present a potentially highly valuable yet underutilized resource in forensic science. Their ubiquitous nature, resilience, and ease of attachment to various surfaces make them excellent indicators in forensic investigations. These attributes allow bryophytes to provide potential crucial links between crime scenes, suspects, and victims.

#### PMI estimation

Bryophytes have been successfully used to estimate the PMI, providing additional data alongside traditional methods. Their growth patterns and species-specific characteristics enable accurate aging of remains, as demonstrated in various case studies. This application is particularly beneficial in cases where other biological markers may not be reliable.

#### Case study evidence

Multiple case reports highlight the diverse applications of bryophytes in forensic investigations. From establishing timelines in homicide and suicide cases to aiding in missing-persons investigations, bryophytes have proven their utility. These cases illustrate how bryophyte evidence can be critical in reconstructing events and providing corroborative data.

#### Increased awareness and training

The number of individuals who are trained to identify microscopic organisms, specifically bryophytes, in the field when investigating a crime are scarce, which potentially limits botanical evidence from being utilized. Efforts should be made to educate forensic professionals and law enforcement agencies about the potential applications of bryophytes in forensic science. Training programs and workshops can enhance the skills required to identify, collect, and analyze bryophyte evidence effectively.

#### Reference databases and herbaria

There are an increasing number of digital and online resources developing reference databases for bryophyte species both regionally and globally. The Consortium of Bryophyte Herbaria (https://bryophyteportal.org/portal/) serves as gateway to plant biodiversity data for mosses, liverworts, and hornworts. These resources will facilitate the comparison of evidence collected from crime scenes with known reference samples, improving the reliability of forensic investigations.

#### Advancements in molecular techniques

The integration of molecular techniques and tools could help to further revolutionize the way that botanical evidence is applied to forensic applications and strengthen conclusions. Environmental DNA, also known as eDNA, has recently emerged and is being used in ecology and conservation efforts [83].

#### Interdisciplinary collaboration

Collaboration between bryologists, forensic scientists, and law enforcement agencies is crucial for the successful application of bryophytes in forensic investigations. Interdisciplinary research projects can further explore the potential of bryophytes and develop new methodologies for their use in forensic science.

#### Broader ecological studies

Expanding ecological studies on bryophytes can enhance our understanding of their growth patterns, environmental interactions, and resilience. This knowledge will contribute to more accurate applications in forensic investigations, particularly in diverse environmental conditions.

#### Policy and protocol development

Developing standardized protocols and guidelines for the collection, preservation, and analysis of bryophyte evidence will ensure consistency and reliability in forensic investigations. These protocols should be integrated into existing forensic botany frameworks to streamline the incorporation of bryophyte evidence.

## Concluding remarks

It is clear that bryophytes present a highly valuable yet potentially underutilized tool in forensic botany. Their ubiquitous nature, remarkable resilience, and their ability to become easily attached to substrates make them excellent indicators within criminal investigations. These characteristics allow bryophytes to provide associations between suspects, victims, and the crime scenes and have proven their potential. There have been numerous case reports that highlight the diverse applications of bryophytes to forensic investigations including but not limited to PMI calculations, homicides, suicides, and missing-persons investigations. These cases illustrate how bryophyte evidence can be critical in reconstructing events and providing corroborative data. This comprehensive review of bryophyte application to forensic science aims to raise awareness and encourage law enforcement to be mindful of the potential of plant fragments, microscopic biotic material, and small plants such as bryophytes as part of their investigative processes. Undoubtedly, there are possible limitations to the applicability of bryophytes to forensic science in terms of species-specific responses to biotic and abiotic factors. For example, different moss species may have varying responses to environmental conditions as well as potential difficulties in preservation and analyzing moss evidence in diverse environments. Yet, physical evidence is often presented in court, even where its value is limited, to corroborate witnesses or other physical evidence. We hope this encourages an increased awareness of bryophytes and similar microscopic plants when undertaking forensic investigation, ensuring that critical plant evidence is not overlooked in the future.

## Note

Voucher specimens seen in figures that are representative, only, of the species outlined in this review. All specimens are deposited in the herbarium at Field Museum (F):

1. *Hypnum cupressiforme*, Daniel C. Eaton, 142, Connecticut, New Haven, USA, https://fm-digital-assets.fieldmuseum.org/593/538/C1065203F.jpg, [C1065203F]

2. *Dicranum scoparium*, C. E. Darigo, 4096, USA, https://fm-digital-assets.fieldmuseum.org/497/309/C1055519F.jpg*,* [C1055519F]

3. *Ceratodon purpureus*, M. M. Cook & M. V. Thayer, s.n., New Hampshire, Kearsage, USA, https://fm-digital-assets.fieldmuseum.org/482/406/C1044845F.jpg, [C1044845F]

4. *Ptychostomum capillare*, I. Schnooberger, 3942, Vermont, Windham, Newfane, Steddon, USA, https://fm-digital-assets.fieldmuseum.org/488/707/C0074109F.jpg, [C0074109F]

5. *Brachythecium albicans*, V. F. Brotherus, n/a, Nylandia, Finland, [C2025573F]

## Discloure statment

None declared.
